# Return to Work After Subcutaneous Transposition of the Extensor Indicis Proprius to Repair Inveterate Ruptures of Extensor Pollicis Longus

**DOI:** 10.3390/jcm14030814

**Published:** 2025-01-26

**Authors:** Gabriele Tamburrino, Giuseppe Rovere, Lucian Lior Marcovici, Filippo Migliorini, Camillo Fulchignoni, Andrea Fidanza

**Affiliations:** 1 Unit of Orthopaedics, Department of Life, Health and Environmental Sciences, University of L’Aquila (IT), 67100 L’Aquila, Italy; gabriele.tamburrino@graduate.univaq.it; 2 Section of Orthopaedics and Traumatology, Department of Clinical Science and Translational Medicine, University of Rome “Tor Vergata”, 00133 Rome, Italy; giuseppe.rovere02@icatt.it; 3 Hand Surgery Unit, Department of Orthopaedics and Traumatology, Fondazione Policlinico Universitario A.Gemelli IRCCS, 00168 Rome, Italy; camillo.fulchignoni2@guest.policlinicogemelli.it; 4 Hand & Microsurgery Unit, Jewish Hospital of Rome, 00186 Rome, Italy; lucian.marcovici@uniroma1.it; 5 Department of Orthopaedic and Trauma Surgery, Academic Hospital of Bolzano (SABES-ASDAA), Via Lorenz Böhler 5, 39100 Bolzano, Italy; migliorini.md@gmail.com; 6 Department of Life Sciences, Health, and Health Professions, Link Campus University, 00165 Rome, Italy

**Keywords:** tendon injuries, extensor tendon, hand surgery, hand injuries, thumb, index, fingers, rehabilitation, engagement in work, factors related to return to work

## Abstract

**Background/Objectives**: An Extensor Pollicis Longus (EPL) subcutaneous rupture is a substantial complication in post-traumatic or degenerative wrist and tendinous lesions. The diagnosis is essentially dictated by a clinical evaluation; in fact, it is characterized by the inability to extend the thumb interphalangeal joint and to retropose the thumb while the hand is resting on a surface. The tendinous transposition using the Extensor Indicis Proprius (EIP) as a donor tendon is a well-known surgical technique performed to restore functional activity to the thumb, and it is preferred for the closer cerebellar network with the thumb itself. However, there is a dearth of clinical results and scientific evidence in the literature. The aim of this study is to evaluate the return-to-work eligibility after an inveterate EPL subcutaneous rupture repaired with a transposition of the EIP. **Methods**: Patients who reported a subcutaneous rupture of the EPL due to rheumatic diseases or who had undergone previous hand or wrist surgery were excluded; however, all patients tested positive for traumatic wrist hypertension. The surgical technique involves three small incisions to achieve tenorrhaphy of the EIP at the distal head of the EPL. Dynamic tests are carried out intraoperatively to verify the tightness and sufficient rigidity of the suture. The objective evaluation involves the range of motion, pinch strength, and power extension of the thumb and the index finger. Patient-reported outcome measures for pain and patient satisfaction include the Numeric Pain Rating Scale and the Disabilities of the Arm, Shoulder, and Hand questionnaire. **Results**: A total of 12 patients were eligible (7 W, 5 M, mean age 56.3 years) and were followed for at least one year. There were no surgery-related complications. After the cast was removed 3 weeks after surgery, patients could extend their thumbs, put them back, and use their index fingers. An immediate improvement in objective and subjective assessments was reported. At 10 weeks, nine patients (75%) returned to full work with no pain and without the aid of rehabilitation; two patients (17%) returned to full work with no symptoms 2 weeks later; and only one patient (8%) with a neurological disease required physical therapy, achieving pain control and restoration of full mobility approximately six months after surgery. **Conclusions**: This surgical technique seems to address satisfactory results in terms of skill recovery and rapid return to work. A tailored rehabilitation program should be implemented for selected patients with neurological conditions that may prolong the adaptation process of the transposed tendon, the coordination, and the independent function of the thumb and index finger.

## 1. Introduction

The Extensor Pollicis Longus (EPL) is a deep muscle at the back of the forearm and hand. It originates from the posterior shaft of the distal ulna and the interosseous membrane. It forms a tendon before crossing the wrist joint, changing the angle at the Lister’s tubercle to insert at the distal phalanx of the thumb [1]. A spontaneous rupture of the EPL is uncommon and mainly reported after degenerative tendinous lesions resulting from synovitis, tenosynovitis, or rheumatoid arthritis [2]. Up to 12.5% of distal radius fractures are stabilized with a volar plate, and screws can lead to extensor tendon ruptures when screws penetrate the dorsal side of the wrist [3,4]. Denman hypothesizes that wrist hyperextension trauma could cause impingement between the radial styloid and the base of the third metacarpal bone, resulting in a partial tendon lesion initially, followed by a total lesion later [5]. Dums considered this lesion to be typical of young drummers in military bands [6]. However, an EPI subcutaneous rupture usually occurs in individuals with rheumatic or post-traumatic disease [7]. In general, microlesions from fractures or sprains of the wrist can cause an EPL rupture, typically at its reflection point around the Lister’s tubercle, where the tension is greatest and the vascularity is at its lowest [8].

Clinically, it is characterized by an inability to extend the thumb’s interphalangeal (IP) joint and reposition it while the hand rests on a surface. The patient usually does not feel pain and usually recognizes the limitations in thumb movement only a few days later, which prompts them to receive a specialist consultation. Ultrasound and magnetic resonance imaging (MRI) can be used as diagnostic tests, although ultrasound has limited iconographic utility, whereas MRI can better comprehend tendon diseases. Fundamentally, the diagnosis is essentially dictated by a clinical evaluation.

Several surgical techniques have been described to repair this injury. Tendon transposition using the Extensor Indicis Proprius (EIP) was developed by Mensch in 1925 [9], used by various authors [10,11,12,13,14,15], and then modified with transposition of the Extensor Carpi Radialis Longus [16,17], the extensor proprius of the fifth finger [18], and even tenodesis of the extensor pollicis brevis [19,20,21]. Other treatments proposed include McMaster’s direct suture [22], the palmaris gracilis graft [23], and thumb IP arthrodesis.

However, the literature lacks clinical results and scientific evidence: there is a lot of heterogeneity about clinical evaluations of the thumb and index after EIP transposition; in one of the first clinical studies [13], the value of the flexion and extension of the thumb, even compared with the contralateral limb, was enough to give an assessment of the result of the surgery. Recently, studies have focused on other objective outcomes, such as assessing the extension and flexion strength of both the thumb and index finger [24,25,26]. A relation between functional outcome and the assessment of strength and ROM with work performance [27] was also studied, but it referred to hand trauma and was not directly related to EPL ruptures restored with EIP transposition.

The aim of this study is to evaluate returning to work and life activities after an inveterate Extensor Pollicis Longus subcutaneous rupture treated by transposition of the Extensor Indicis Proprius.

## 2. Materials and Methods

From 2021 to 2023, patients with a subcutaneous inveterate rupture of the Extensor Pollicis Longus were prospectively recruited at an Upper Limb Center in central Italy. Patients with rheumatic diseases (such as rheumatoid arthritis or psoriatic arthritis) and patients who had undergone previous wrist or hand surgeries were excluded. On clinical examination, all patients presented an inability to extend the distal phalanx of the first finger with their palm resting on a flat surface; the absence of retropulsion is a hallmark of an EPL injury. None of them showed limitations in the ROM of the second finger, from maximum active flexion to maximum active extension.

All patients were fully informed, clearly and comprehensively, of the proposed surgical procedure and possible risks. Patients were treated according to the ethical standards expressed in the Declaration of Helsinki and were asked to read, understand, and sign the informed consent form. All patients gave consent for their data and photos to be published for scientific purposes. The Young European Hand Surgeons (YEHS) committee approved the research.

### 2.1. Surgical Technique

**Figure 1:**
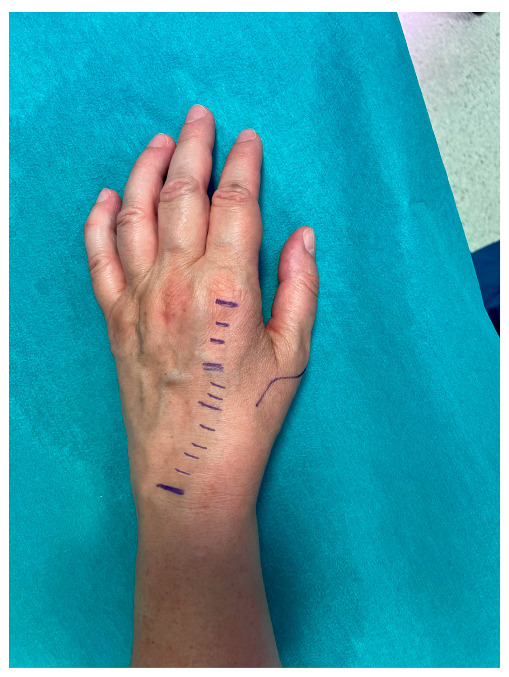
Surgical technique incisions. The dotted lines follow the tendon’s path.

All procedures were performed under brachial plexus anesthesia by the same surgeon. The tourniquet was applied in all procedures before incision and removed before wound closure. In this technique, only three small incisions are needed to expose the EIP [28,29,30,31,32]: (1) a smaller transverse incision on the head of the second metacarpal bone (MC) to isolate the EIP, which is located ulnarly to the Extensor Index Digitorum Communis (EICD). It is dissected and brought out of the first incision while the distal stump is sutured to the extensor cuff. (2) A second transverse incision was made at the base of the second MC to follow the course of the tendon and keep it taut for the third incision. (3) A transverse incision, distal to the Lister’s tubercle, is performed. At this point, the distal head of the EIP is dislocated subcutaneously, emerging from the last incision made (Figure 1).

**Figure 2:**
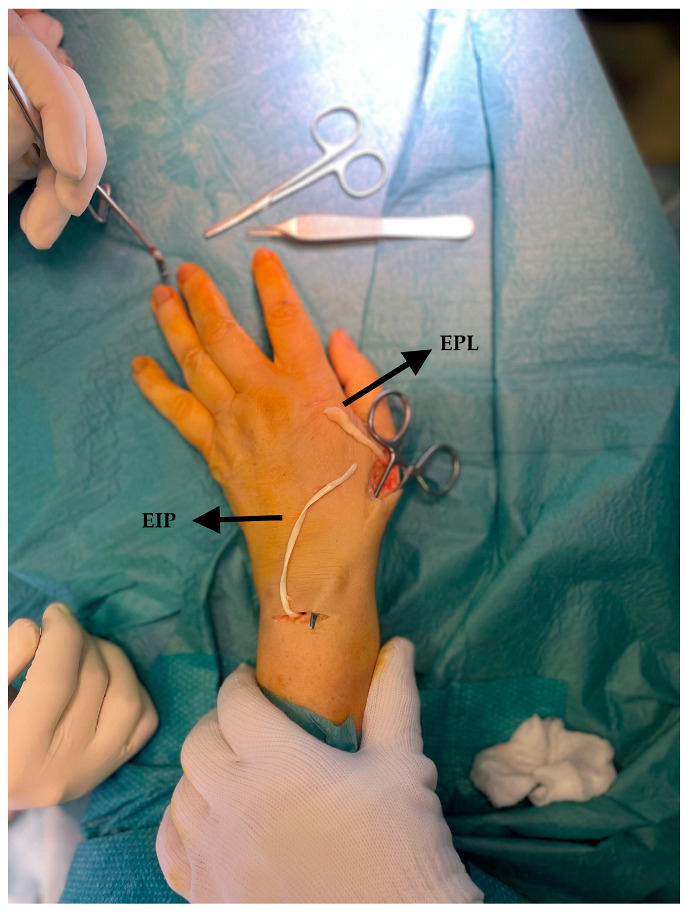
Subcutaneous transposition of the Extensor Indicis Proprius (EIP) to obtain tenorrhaphy at the proximal head of the Extensor Pollicis Longus (EPL).

**Figure 3:**
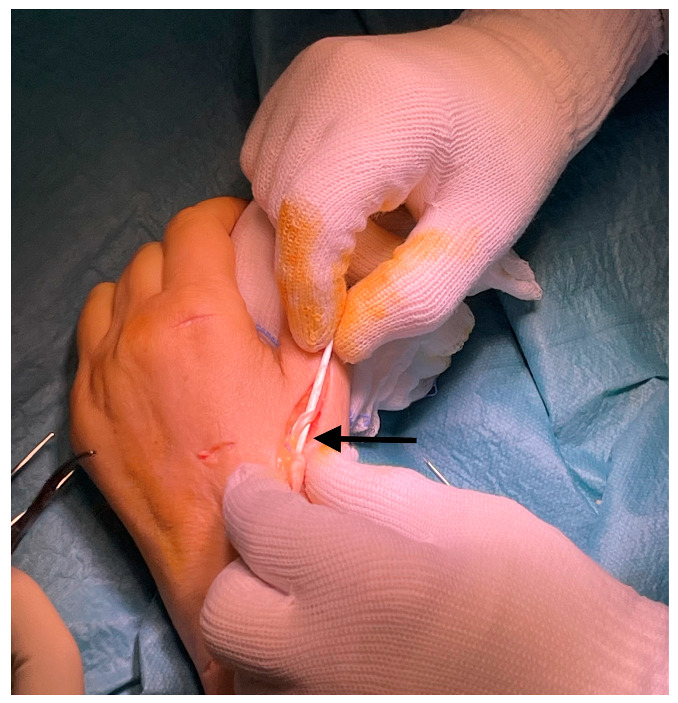
Side-to-side tenorraphy through a buttonhole (arrow) created to pass the EIP into the EPL before fixing it to the correct tension.

Finally, once the donor tendon has been isolated, an arched incision is made at the level of the neck and the diaphysis of the first MC to detect the EPL tendon, which will already appear distally with hypertrophic scarring and will be easily identifiable and dislocatable in a disto-proximal direction (Figure 2). The free stump of the EIP is passed subcutaneously until it emerges at the neck of the I MC. The authors use only the extensor retinaculum as a pulley, without constructing additional ones, nor passing the tendon under the Extensor Carpi Radialis Longus to avoid adhesions and pain in sliding during flexion-extension of the thumb. At this point, the EIP is connected to the EPL through a buttonhole created on the latter (Figure 3) and suturing the terminal stumps with Nylon 3/0, which provides both an efficient transmission of force and high resistance in the suture, as described by Frieden in his side-to-side tenorrhaphy [33,34,35,36].

**Figure 4:**
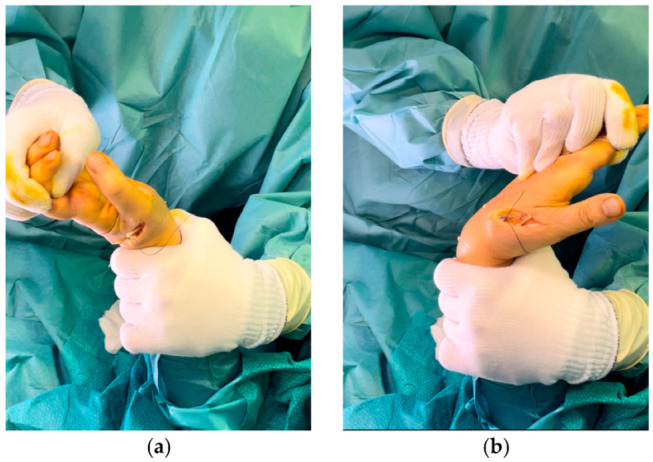
Dynamic test before performing the definitive tenorrhaphy: while the wrist is extended and held in a radial position, the IP and MC-P of the thumb will flex (**a**), while, when the wrist is flexed, these joints will extend (**b**).

**Figure 5:**
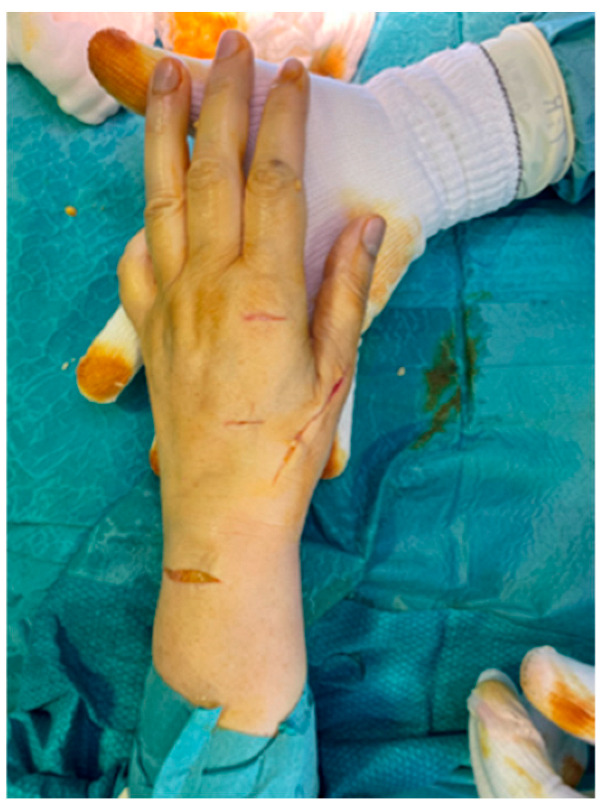
Closed incisions.

Before completing the tenorrhaphy, dynamic intraoperatively tests are performed to verify the tightness and sufficient rigidity of the suture: while the wrist is kept in a radial position, extending it will cause flexion of the IP and MC-P of the thumb (Figure 4a), while, when the wrist is flexed, these joints must extend (Figure 4b). It may be useful to use temporary stitches to perform dynamic maneuvers and to possibly remove and reposition them until the tension and excursion are satisfactory. Finally, the tendon stump is re-tied to close the loop, and some stitches are placed with Nylon 3/0 and 4/0 to complete the tenorrhaphy. Lastly, after removing the tourniquet, careful hemostasis, abundant washing, and suturing conclude the operation (Figure 5).

### 2.2. Post-Operative Care

A plaster glove, including the wrist and first finger in extension, is positioned for 3 weeks. The suture is removed, and the patient is asked to wear a night splint as a thumb stabilizer only during the night for 2 weeks; gradual and progressive resumption of active mobilization of the metacarpophalangeal joint of the first finger is permitted. No physiotherapy rehabilitation is recommended unless complications arise.

### 2.3. Objective Evaluation

The active and passive ranges of motion (ROMs) were measured with a universal 360° goniometer in which the fulcrum was dorsally placed on the first MC-P joint [37]. Pinch strength was measured using a Jamar dynamometer (Asimow Engineering Co., Santa Monica, CA, USA) placed between the radial side of the index finger and thumb, and the patient was instructed to pinch as hard as possible, as described in the literature [38], with the percentage being compared with the non-operated side; additionally, an extensometer (Penny and Giles Transducers, RADWARD LTD, Hemel Hempstead, UK) was used to measure the power of the extension of the thumb and the index finger independent of the position of the other fingers to avoid distortion of the force due to adhesions. Therefore, the extension strength of the I and II fingers was measured both during the flexion of the III, IV, and V fingers (independent extension) and during the extension of all fingers (dependent extension) at 10 weeks after the surgery and after one year. The same instrument was used on all patients and calibrated at regular intervals. All the measurements were repeated three times and the average was calculated.

### 2.4. Personal/Subjective Evaluations

The patient’s pain was evaluated using the Numeric Pain Rating Scale (NPRS) [39], a segmented numerical version of the Visual Analog Scale (VAS) [40]. Patient satisfaction was evaluated using a Disabilities of the Arm, Shoulder, and Hand (DASH) questionnaire [31]. All of these functional values were compared to those of the contralateral limb.

Additional clinical assessments were conducted at 6 and 10 weeks to assess the recovery of life skills and return-to-work eligibility, defined as the first time a patient-reported returning to work at at least 50% of their original hours per week, as specified in their contract. Future follow-ups were performed at 6 months and 1 year after surgery.

## 3. Results

A total of 12 consecutive patients were eligible, including five men and seven women, with a mean age of 56.3 years (min 42–max 65). All patients enrolled reported a previous contusive or sprain trauma to the wrist; six of them had a fracture of the distal radius that was conservatively treated with plaster. The occupations of the sample were as follows: teachers (2), employees (2), crossfit coaches (1), tennis players (1), engineers (1), farmers (3), and physicians (2). The time from the onset of symptoms to the day of surgery went from one week to five weeks (mean 2.8 weeks). Depending on whether the procedure was performed in the morning or afternoon, five patients were discharged in the evening and seven were discharged the next day.

There were no surgery-related complications. Once the cast was removed, patients could extend the thumb, put it back, and use their index finger, which is why patients were encouraged to move actively from the beginning. After 6 weeks, 91.7% of patients showed no delay in flexion and extension of the interphalangeal joint of the thumb and index finger, with good pain control.

The objective and subjective outcomes were overall satisfactory. As reported in Table 1, the pain symptom changed from an average of 6.2 ± 1.65 in all patients before surgery to 1.9 ± 1.26 at the last follow-up. The thumb ROM also improved from 48.7 ± 14.2° to 70.3 ± 13.4° of thumb flexion at the MC-P joint, recovering a ROM comparable to the contralateral one. The DASH score decreased from 70.93 ± 9.48 to 11.40 ± 12.01. Pinch strength and finger extension at the final follow-up are reported in Table 2.

The primary outcome measures were time to a full return to work (the same number of hours worked before the injury) and the duration of complaints in weeks. Having complaints was defined as “having pain or physical limitations to the extent that it affects several daily activities”, with the date of surgery being chosen as the starting point for the period of absence from work: at 10 weeks, nine patients (75%) returned to full work with no pain and ROM gaps in the thumb and index finger; two patients (17%) returned to full work with no symptoms 2 weeks later (in fact, one tennis player and one physician maintained pain during a specific high-demand skill, such as holding a racket during training or holding and cutting with scissors); only one farmer (8%) required physical therapy to achieve good pain control and restore a full ROM and thumb strength, and this occurred approximately six months after surgery.

## 4. Discussion

According to the main findings of the present study, an Extensor Indicis Proprius transposition aimed at restoring thumb extensor function is a safe and reliable procedure with no particular complications or surgical difficulties. It ensures a rapid return to work and previous duties.

When the EPL ruptures, the phalanges fall off and the thumb becomes unable to extend and lift. This condition not only makes the subject unfit for manual labor but also worsens the standard of living for everyone. Indeed, if the initial stage of the injury involves the inability to elevate the interphalangeal joint, this disease will become chronic and ultimately cause stiffness and obstruction in the IP’s flexion, resulting in a “mallet finger” deformity, meaning that the fingertip becomes chronically bent and appears crooked, giving the finger the appearance of a hammer or mallet. Therefore, surgery is mandatory to restore thumb functionality.

It is widely accepted that direct suturing in these injuries should not be performed due to the loss of tendon substance from friction, which may result in sutures arriving at a tendon reflection point with little assurance of mechanical integrity. Further shortening of the proximal end may occur due to myostatic contracture, particularly if the presentation is delayed. Similarly, tendon grafts are often discarded due to the need for dual surgical approaches, both volar and dorsal, at the wrist and the requirement for double tendon sutures [23].

Tendon transpositions have emerged as the preferred surgical approach due to their simplicity, reliable outcomes, and extensive documentation in the literature. While various transposition techniques have been described, each have their limitations. For instance, the tenodesis of the extensor pollicis brevis does not provide adequate glide or strength for thumb extension [13]. Similarly, the Extensor Digiti Quinti Proprius transposition, first proposed and predominantly applied by Verdan [16], has shown poor sliding and insufficient extension strength. Transposition of the Extensor Carpi Radialis Longus has also been reported with satisfactory results, but the authors caution against its use as it may destabilize the wrist’s radioulnar balance. This technique may be considered only for selected cases where EIP transposition is not viable, such as when the EIP has already been used for other tendon transfers [16,17]. However, EIP transposition remains the most widely used and historically supported technique [9,10,11,12,13,14,15].

EIP transposition offers several advantages. Its anatomical proximity and redundancy for index finger extension make it an ideal donor tendon. Furthermore, the EIP and EPL are synergistic, as extensions of the index finger and thumb interphalangeal joint often occur together during functional tasks. This synergy reduces the need for extensive re-learning after the transfer. Anatomical studies have demonstrated that the EIP is distinct from the Extensor Indicis Communis (EICD) tendon in regard to both position and function. The EIP is the more ulnarly positioned of the two index finger extensor tendons in 96.5% of populations and exhibits functional independence due to its deeper location and isolated muscle belly. EIP’s muscle belly is the sole/only/unique that enters the fourth compartment, lying below the retinaculum and deeper than the extensor communis tendons [41]. Upon careful intraoperatively visual inspection, tendon size certainly does not help distinguish EIP from EICD: the tendon circumference at the distal edge of the extensor retinaculum averaged 9.3 mm (±1.7 mm) for EICD and 11.1 mm (±2.7 mm) for EIP; however, the tendon circumference at the MC-P index joint was measured to be 11.0 mm (±1.7 mm) for EICD and 10.6 mm (±2.1 mm) for EIP. There were no significant differences in tendon circumferences between genders, but, overall, tendon circumferences were smaller for females than for males [41].

A technique to distinguish EIP from EICD is instead dynamic: the association between the extension of the index finger and the simultaneous flexion of the III, IV, and V fingers (the “gun sign”) involves the move in the ulnar direction of the EICD due to the traction of the tendon adhesions. On the contrary, the EIP remains in its natural position and becomes visible and isolated thanks to the migrated EICD tendon [42].

An important factor for the success of the transfer from EIP to EPL is the optimal tension of the tendons before performing the definitive tenorrhaphy in relation to the position of the thumb and wrist. Previous work has established that the tension on the transferred tendon should be strong while still allowing for thumb flexion with the wrist in a neutral position [32]. While other authors recommend performing tenorrhaphy with the thumb in full extension and the wrist in a neutral position, this will result in an initial loss of thumb flexion during the first few months and, therefore, a lower grip and pinch strength, regaining it at 1 year follow-up, when the thumb tip will reach adequate flexion and extension function superimposable on the healthy side [43]. Current authors prefer to fix the final tension by performing dynamic tests: after radial flexion of the wrist, if the surgeon extends it, he will cause a flexion of the phalanges, while, when moving the wrist in flexion, the thumb will experience an immediate extension. This technique is allowed in order to ensure an adequate extension of the distal phalanx on the same level as the healthy side while offering an immediate recovery of thumb flexion to ensure an adequate resumption of daily activities.

Current findings align with the literature, suggesting that the type of job significantly influences the return to work following wrist and hand injuries [44,45,46]. A common criticism of EIP transposition is the potential for residual extension deficits in the index finger, which could limit high-demand tasks requiring a significant index finger ROM and strength. However, these deficits are likely due to scarring around the extensor hood rather than an actual loss of extension power [47,48]. This was corroborated by several authors [26,49,50] who reported favorable outcomes with preserved index finger strength and extension after EIP transposition. Similarly, the presented cohort observed no significant limitations in thumb or index finger function, with all patients achieving satisfactory outcomes for their occupational demands.

Although women appeared to take longer to resume their duties, relevant gender differences in return-to-work timelines were noted in the present study. This finding contrasts with previous papers, including those by Chang et al. [51], Lee et al. [52], and Skov et al. [53], which found no significant gender-based differences in recovery times. However, these studies were retrospective, relying on medical records, which may have influenced their findings. In the present study, grip strength and function measures correlated strongly with return-to-work outcomes, supporting the conclusions of S.K. Wahi Michene [27], who emphasized how crucial it is to include assessments of grip strength and function when evaluating the recoveries from hand trauma. Furthermore, the assessment of strength using the value of the non-operated side was important in setting the required strength of the muscles involved [54] and, subsequently, a starting point for returning to work after this specific kind of hand injury. In future studies it would be interesting to integrate advanced biomechanical tests to validate functional recovery metrics.

Furthermore, in regard to the impact of hand and wrist injuries on physical and mental health, these injuries can be more significant than knee, hip, and head trauma according to the intensity of injuries occurring each year [55]; they can also lead to high health-care costs and prolonged time off the work, with rehabilitation potentially adding even more time to such absences [56]. While sophisticated cost analysis systems have been developed for elective orthopedic surgeries, it is more difficult to have a precise evaluation of direct and indirect costs for traumatology [57]. Indirect costs, which are typically covered by the individual, family, community, and employer, and which are related to morbidity and mortality, are more challenging to measure than direct costs. To expedite recovery from hand trauma, enrolling in a rehabilitation program can help people to return to a high standard of personal and professional life. However, the cost of rehabilitation may differ depending on the health and economic policies of each country [58]. In particular, the management of a rehabilitation program for each individual is planned around a wide range of objective measures regarding impaired function while also taking into account the functional needs and goals of the patients [59]. Notably, 11 out of 12 patients the presented cohort did not require formal physiotherapy to regain occupational function, nor did they feel the need for it, because they were able to move normally after the cast was removed. This indicates that the motor cortex and their network are identical, which is likely why thumb and index coordination and movement independence do not require adaptive time. Only one patient, a farmer with a pre-existing neurological condition, needed a tailored rehabilitation program, and they achieved full recovery after six months. This underscores the importance of considering neurological conditions that may prolong the adaptive process of the transposed tendon, necessitating specialized hand physiotherapists to optimize outcomes and a rehabilitation program focused on the recovery of proprioception and the individual use of the two fingers with fine and precision movements.

One of the main limitations of this study is the small sample size, which is consistent with other reports in the literature. Although the rarity and delayed diagnosis of these injuries constrain the ability to study a large, consecutive, and prospective sample, future randomized clinical trials, possibly with larger populations, should be performed to understand whether variations in surgical techniques might influence the results.

Given the retrospective nature of the study design, a formal power analysis was not performed prior to data collection. The study relied on existing data, and the sample size was determined by the availability of relevant records that met the inclusion and exclusion criteria. While this approach may limit the ability to pre-determine statistical power, it reflects the real-world constraints often associated with retrospective observational research. The study’s findings should therefore be interpreted with caution, recognizing this limitation.

On the contrary, the main strength of this study is that it is the first to specifically investigate return-to-work outcomes following EIP transposition, providing valuable insights into this under-researched area. Furthermore, the use of a minimally invasive surgical technique allowed for immediate patient discharge and the reducing of direct costs, while the use of a tendon donor coming from the same motor cortex as the injured tendon eliminated the need for rehabilitation for all patients without previous neurological pathologies while also reducing indirect costs to society.

## 5. Conclusions

The delayed manifestation of function impairment following an EPL injury often results in inveterate cases by the time patients seek surgical intervention. Tendon transposition of EIP has proven to be a reliable and well-documented solution, offering patients a swift return to work and daily activities. A thorough evaluation of the patient’s medical history is essential in regard to optimizing outcomes, particularly in terms of identifying any underlying neurological conditions. A tailored rehabilitation program should be implemented for selected patients to ensure proper coordination and independent thumb and index finger functions.

## Figures and Tables

**Table 1:** Evaluation of the NPRS, DASH, and ROM before the intervention and at the one year follow-up. The results are expressed as mean.

Variables	Pre-Operative	Post-Surgery
NPRS	6.2	1.9
DASH	70.93	11.40
ROM (thumb)	48.7°	70.3°

**Table 2:** Outcome of the sample one year after tendon transfer. The pinch strength is expressed as a percentage of the unoperated side.

Work (Gender)	Time to Surgery	IEP Thumb	DEP Thumb	IEP Index	DEP Index	Pinch Strength (%)	Return to Work
Teacher (F)	1 w	1.9 kg	1.0 kg	0.8 kg	0.8 kg	86	10 w
Teacher (F)	3 w	1.8 kg	1.1 kg	0.8 kg	0.7 kg	85	13 w
Employee (F)	4 w	2.0 kg	1.2 kg	0.9 kg	0.9 kg	84	10 w
Employee (F)	2 w	1.9 kg	1.2 kg	0.7 kg	0.7 kg	84	10 w
Crossfit trainer (M)	5 w	2.3 kg	1.7 kg	1.0 kg	1.0 kg	88	10 w
Tennis player (M)	4 w	2.1 kg	1.5 kg	0.9 kg	0.8 kg	86	13 w
Engineer (F)	3 w	2.0 kg	1.4 kg	0.8 kg	0.8 kg	85	10 w
Farmer (M)	4 w	1.6 kg	0.9 kg	0.8 kg	0.6 kg	80	24 w
Farmer (M)	1 w	2.1 kg	1.2 kg	1.0 kg	0.9 kg	84	10 w
Farmer (F)	4 w	2.0 kg	1.4 kg	0.8 kg	0.8 kg	86	10 w
Physicians (M)	2 w	2.0 kg	1.1 kg	0.8 kg	0.9 kg	86	10 w
Physicians (F)	1 w	1.9 kg	1.2 kg	0.7 kg	0.8 kg	85	10 w

IEP: independent extension power (measured during the flexion of the III, IV, and V fingers); DEP: dependent extension power (measured during the extension of all fingers).

## Data Availability

The datasets used and/or analyzed during the current study are available from the corresponding author on reasonable request.

## Abbreviations

The following abbreviations are used in this manuscript:

EPL	Extensor Pollicis Longus
EIP	Extensor Indicis Proprius
IP	Interphalangeal
MRI	Magnetic Resonance Imaging
YEHS	Young European Hand Surgeons
MC	Metacarpal Bone
EICD	Extensor Index Digitorum Communis
MC-P	Metacarpophalangeal Joint
NPRS	Numeric Pain Rating Scale
VAS	Visual Analog Scale
DASH	Disabilities of the Arm, Shoulder, and Hand
ROM	Range Of Motion
